# Automatic diagnosis of imbalanced ophthalmic images using a cost-sensitive deep convolutional neural network

**DOI:** 10.1186/s12938-017-0420-1

**Published:** 2017-11-21

**Authors:** Jiewei Jiang, Xiyang Liu, Kai Zhang, Erping Long, Liming Wang, Wangting Li, Lin Liu, Shuai Wang, Mingmin Zhu, Jiangtao Cui, Zhenzhen Liu, Zhuoling Lin, Xiaoyan Li, Jingjing Chen, Qianzhong Cao, Jing Li, Xiaohang Wu, Dongni Wang, Jinghui Wang, Haotian Lin

**Affiliations:** 1 School of Computer Science and Technology, Xidian University, No. 2 South Taibai Rd, Xi’an, 710071 China; 2 School of Software, Xidian University, No. 2 South Taibai Rd, Xi’an, 710071 China; 3 State Key Laboratory of Ophthalmology, Zhongshan Ophthalmic Center, Sun Yat-sen University, Xian Lie South Road 54#, Guangzhou, 510060 China; 4 School of Mathematics and Statistics, Xidian University, Xi’an, 710071 China

**Keywords:** Imbalanced ophthalmic images, Pediatric cataracts, Lens automatic localization, Cost-sensitive, Deep convolutional neural network

## Abstract

**Background:**

Ocular images play an essential role in ophthalmological diagnoses. Having an imbalanced dataset is an inevitable issue in automated ocular diseases diagnosis; the scarcity of positive samples always tends to result in the misdiagnosis of severe patients during the classification task. Exploring an effective computer-aided diagnostic method to deal with imbalanced ophthalmological dataset is crucial.

**Methods:**

In this paper, we develop an effective cost-sensitive deep residual convolutional neural network (CS-ResCNN) classifier to diagnose ophthalmic diseases using retro-illumination images. First, the regions of interest (crystalline lens) are automatically identified via twice-applied Canny detection and Hough transformation. Then, the localized zones are fed into the CS-ResCNN to extract high-level features for subsequent use in automatic diagnosis. Second, the impacts of cost factors on the CS-ResCNN are further analyzed using a grid-search procedure to verify that our proposed system is robust and efficient.

**Results:**

Qualitative analyses and quantitative experimental results demonstrate that our proposed method outperforms other conventional approaches and offers exceptional mean accuracy (92.24%), specificity (93.19%), sensitivity (89.66%) and AUC (97.11%) results. Moreover, the sensitivity of the CS-ResCNN is enhanced by over 13.6% compared to the native CNN method.

**Conclusion:**

Our study provides a practical strategy for addressing imbalanced ophthalmological datasets and has the potential to be applied to other medical images. The developed and deployed CS-ResCNN could serve as computer-aided diagnosis software for ophthalmologists in clinical application.

**Electronic supplementary material:**

The online version of this article (10.1186/s12938-017-0420-1) contains supplementary material, which is available to authorized users.

## Background

Eye diseases present great challenges and are serious threats to human health and quality of life [1]. Ophthalmic imaging technologies play an important role in diagnosing eye diseases [2–4]. Many computer-aided diagnosis methods can achieve satisfactory performance when the sample distribution is roughly uniform between different classes [5–8]. However, imbalanced datasets are inevitable in a variety of medical data analysis situations [6, 8–11], which causes the existing classifiers to exhibit a high false negative rate (FNR) or false positive rate (FPR). False-positive results can cause undue worry, economic burden and waste of medical resources, whereas false-negative misclassifications can lead to delayed treatment onset, cause poor treatment outcomes and hinder the use of artificial intelligence technology for diagnosis. In particular, high FNR and FPR rates deter such applications from being used to benefit people far away from clinics. Therefore, it is imperative to explore a feasible and efficient strategy to address the problem of imbalanced ophthalmic image datasets to achieve higher-performance of computer-aided diagnostic systems.

Retro-illumination images are an inevitable and typical imbalanced dataset in the clinical diagnosis of eye diseases [10, 12, 13] such as pediatric cataracts and posterior capsular opacification (PCO). First, the number of positive samples requiring Nd-YAG (neodymium-doped yttrium aluminum garnet) laser surgery in retro-illumination images is much smaller than the number of negative samples [14]. Second, it is difficult to add additional positive sample images because pediatric cataract images are limited [15, 16]. Third, the high FNR caused by the imbalanced dataset leads to delays in treatment onset, Obstacles to vision development, irreversible amblyopia and even severe vision loss [17]. Therefore, exploring and resolving the classification problems caused by imbalanced retro-illumination image datasets will effectively improve therapeutic procedures for eye diseases. In addition, this study provides an important reference for studies of other imbalanced medical datasets, smoothing the path for the further use of artificial intelligence in clinical applications.

Generally, two types of approaches, namely, data leveling [18–20] and algorithm levelling [9, 21, 22] are employed to address the imbalanced datasets problem. Over- or down-sampling methods used at the data level attempt to balance the majority and minority class proportions by data resampling to address the imbalanced problem. However, this approach can easily lead to redundant or missing information and thus affect the classification performance [20, 21, 23]. By contrast, the cost-sensitive approach using algorithm leveling has a distinct advantage because it makes full use of the original data [9, 21, 22]. Meanwhile, deep convolutional neural network (CNN) models have demonstrated extraordinary performance in medical image recognition tasks [24–29]. In this study, we combine a representative deep learning CNN (deep residual network [30]) and a cost-sensitive data-balancing method to present an effective cost-sensitive residual CNN (CS-ResCNN) for the ophthalmic imbalanced dataset problem. By using a grid-search analysis procedure, we demonstrate the robustness and effectiveness of the CS-ResCNN. Finally, we develop and deploy a web-based computer-aided diagnosis (CAD) software based on our proposed method for patients and ophthalmologists in clinical application.

## Methods

### Dataset

Retro-illumination images were obtained from the Childhood Cataract Program of the Chinese Ministry of Health (CCPMOH) [31] of the Zhongshan Ophthalmic Center at Sun Yat-sen University, one of the largest eye hospitals in China [32]. The dataset included 2705 images, of which 735 positive samples represented patients suffering from serious PCO that required ND. YAG-laser surgery, and 1970 negative samples depicted manageable PCO patients. Each image was examined, discussed and labeled by three experienced ophthalmologists.

### Evaluation metrics

Quantitative indicators were employed to assess the performance of our proposed CS-ResCNN compared with four conventional features, two classifiers and three data-level methods. The four conventional evaluation indicators (accuracy, sensitivity, specificity and precision) were calculated as follows.

1 $$ Accuracy = {{(TP + TN)} \mathord{\left/ {\vphantom {{(TP + TN)} {(TP + FN + TN + FP)}}} \right. \kern-0pt} {(TP + FN + TN + FP)}} $$

2 $$ Sensitivity\,(Recall) = {{TP} \mathord{\left/ {\vphantom {{TP} {(TP + FN)}}} \right. \kern-0pt} {(TP + FN)}} $$

3 $$ Specificity = {{TN} \mathord{\left/ {\vphantom {{TN} {(TN + FP)}}} \right. \kern-0pt} {(TN + FP)}} $$

4 $$ Precision = {{TP} \mathord{\left/ {\vphantom {{TP} {(TP + FP)}}} \right. \kern-0pt} {(TP + FP)}} $$

where *TP*, *FP*, *TN* and *FN* represent the numbers of true positives, false positives, true negatives and false negatives, respectively.

We further applied the *F1*-*measure* (Eq. 5), the *G*-*mean* (Eq. 6), the receiver operating characteristic (ROC) curve, the precision–recall (PR) curve, and the area under ROC curve (AUC) to evaluate our classifier comprehensively under the imbalanced dataset scenario [9, 20–22]. Because the *F1*-*measure* and *G*-*mean* [33, 34] simultaneously considers the accuracy for both the positive and negative classes, their values will be very low when the classifier underemphasizes the minority class and overemphasizes the majority class.

5 $$ F1{-}measure = {{(2*{\rm Recall}*{\rm Precision})} / {({\rm Recall} + {\rm Precision})}} $$

6 $$ G{-}mean = \sqrt {({{TP} / {(TP + FN))}}*({{TN} / {(TN + FP))}}} $$

The ROC curve depicts the true positive rate (sensitivity) and false positive rate (1-specificity); the PR curve depicts the precision and recall. The larger AUC is, the better the classification performance is. We adopted fivefold cross-validation (CV) [35] to obtain the mean values and standard deviations of these evaluation indicators to fairly compare their performance. In detail, the dataset is divided into five approximately equal-sized sub-datasets, and the positive samples are divided equally in each sub-dataset.

### Overall diagnosis framework for imbalanced images

**Fig. 1:**
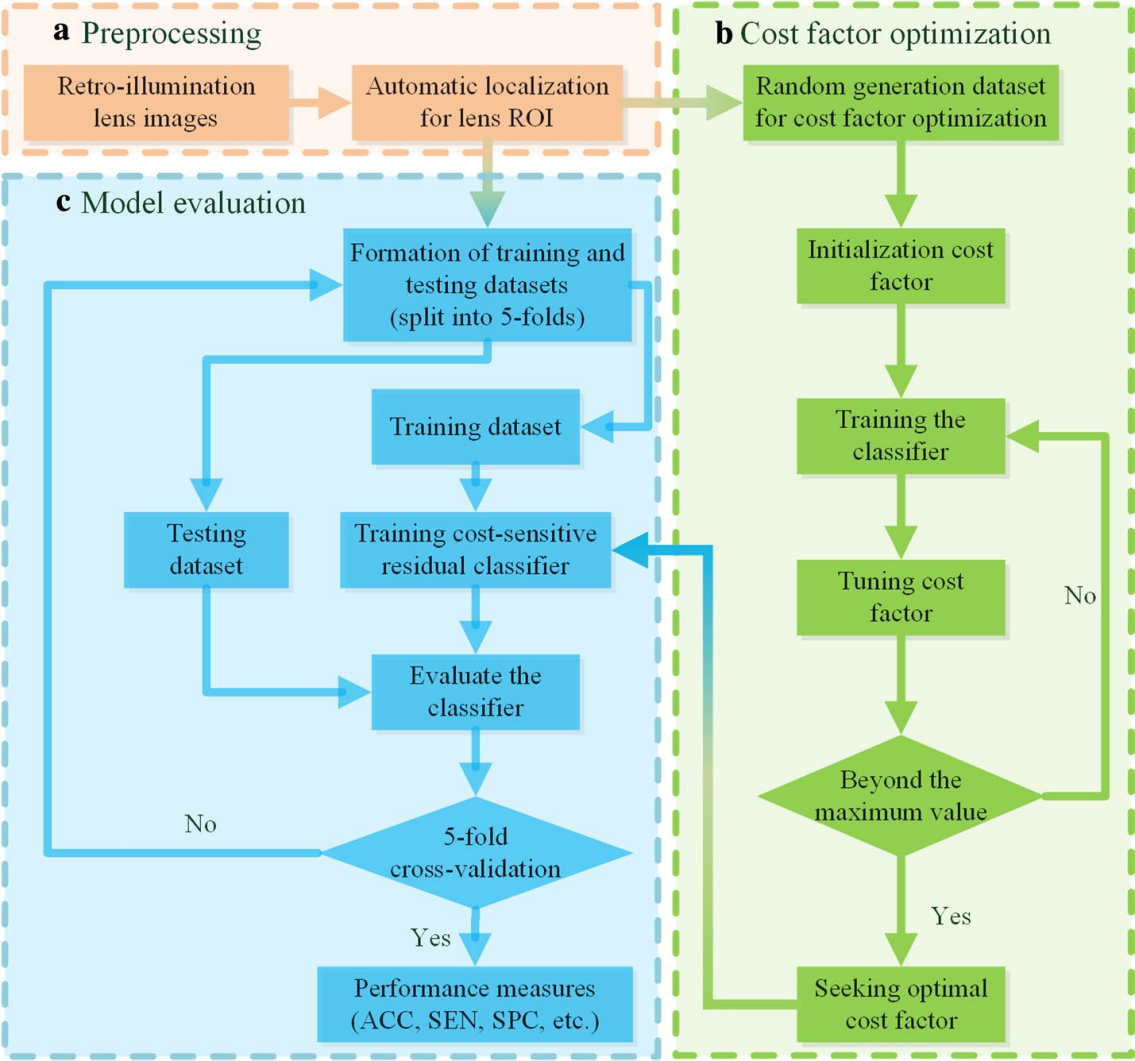
The overall diagnosis framework for imbalanced ophthalmic images. **a** The automatic localization module for lens ROI. **b** The cost-factor optimization module for the CS-ResCNN method. **c** The CS-ResCNN development and evaluation module. ROI, region of interest; CS-ResCNN, cost-sensitive residual convolutional neural network

As shown in Fig. 1, the overall diagnosis framework primarily consists of three modules: automatic localization for lens ROI, cost-factor optimization for the CS-ResCNN model, and CS-ResCNN development and evaluation.

PCO occurs in the lens area, accompanied by strong noise from nearby regions such as the iris and sclera. The boundary of the crystalline lens is an approximately circle in the original retro-illumination images. This characteristic provides a basis for crystalline lens detection. Canny detection and Hough transformation are very suitable for this kind circle detection. We employed two consecutive procedures, Canny detection and Hough transformation [36, 37], to automatically localize the lens region in the original retro-illumination lens images (the red section in Fig. 1a). Then, we created a retro-illumination lens images dataset and resized all cropped regions to 128 × 128, which is sufficiently large to retain the whole pupil but small enough to exclude noisy areas around the pupil area. Resizing the images to the uniform size does not affect the final classification results. Because the ophthalmologists measured the severity of the eye diseases according to the relative opacity location, area and density of lesions in the lens, which do not change in the scaled images.

After obtaining the lens ROI, we randomly selected four-fifths of the cropped images to form a training dataset; the remaining fifth functioned as the testing dataset. By adjusting the cost factor, we trained multiple classifiers to find a suitable cost factor (the green section in Fig. 1b). Finally, the datasets were randomly divided into five parts of approximately equal size, and adopted fivefold cross-validation (CV) to evaluate the performance of the CS-ResCNN model (the blue section in Fig. 1c).

### CS-ResCNN model

**Fig. 2:**
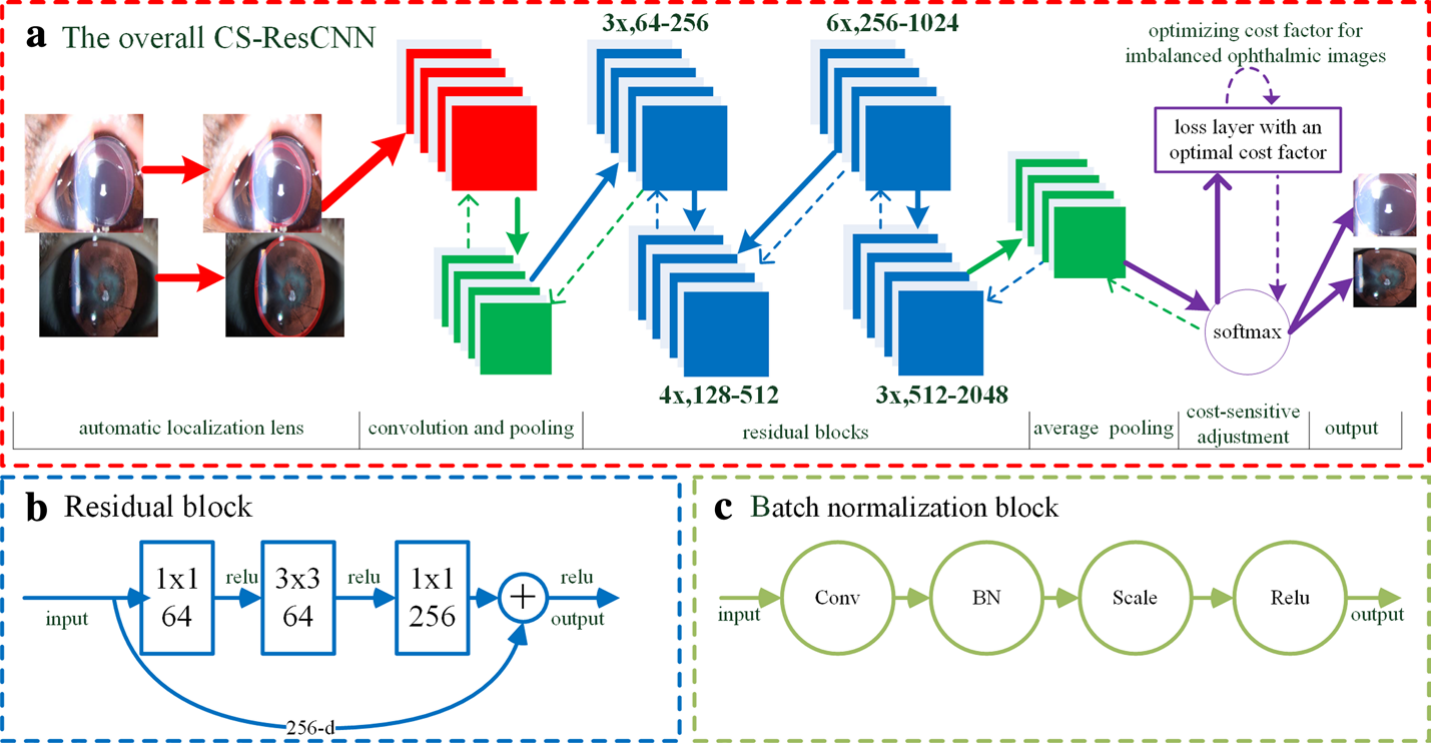
The architecture of the CS-ResCNN method. **a** The overall layers and connections of the CS-ResCNN model consisting of convolution layers, a max-pooling operation and 16 residual blocks, indicated by the red, green and blue rectangles respectively, followed by softmax and cost-sensitive adjustment layers. **b** One unfolded residual block is presented. **c** The BN and scale operations are presented. CS-ResCNN, cost-sensitive residual convolutional neural network; BN, batch normalization; Conv, convolution operation; ReLU, rectified linear unit

Recently, researchers have begun to exploit deeper CNN models to achieve performance improvements in the challenging ImageNet competition [30, 38, 39], which has greatly benefited many nontrivial image recognition applications [24–26, 28, 40]. Inspired by these advanced technologies, we employed an ultra-deep residual CNN network (ResCNN) with 50 layers combined with a cost-sensitive method. As shown in Fig. 2a, the overall architecture of the CS-ResCNN consists of convolutional layers, max pooling operations, residual blocks, batch normalization (BN) blocks [38], softmax cost-sensitive adjustment layers, non-saturating rectified linear units (ReLUs) [41], and data augmentation technology. All of the previous layers are used to extract multidimensional and high-level features from the raw input image, except for the last softmax cost-sensitive adjustment layer which is applied for classification and cost-sensitive adjustment. These essential technologies used in the CS-ResCNN method are briefly introduced in the following sections.

### Residual blocks

As shown in the blue section of Fig. 2a, the residual blocks are employed to avoid the degradation problem in which the accuracy on the training dataset tends to saturate and then to degrade rapidly as the network increases in depth [30, 42]. Each residual block was unfolded into three “bottleneck” building blocks in series where the inputs and the outputs are directly connected. For example, “3x, 64–256” represents three identical residual blocks where the sizes of the input and output feature maps are 64 and 256 respectively (Fig. 2b). There are 16 residual blocks in the whole network, each of which consists of three convolution operations and nonlinear transformations. In other words, the full set of residual blocks is made up of 48 layers. Using the residual technology, the ultra-deep network can be employed to further enhance recognition rates and accelerate convergence.

### Batch normalization and scaling operation

BN blocks [38] are another key technology that addresses the problems of vanishing and exploding gradients which seriously hinder CNN convergence. As shown in Fig. 2c, each complete BN block primarily contains a BN and a scaling operation situated between a convolutional layer and a ReLU in Fig. 2b. As shown in Fig. 2b, each residual block consists of three BN and scaling operations. The BN and scaling operations are defined in Eqs. 7–10, respectively, where *m*, *x*_*i*_, $\hat{x}_{i}$, *y*_*i*_, *μ*_*m*_, $\sigma_{m}^{2}$, *γ*, and *β* represent the mini-batch size, the *i*-th value of input *x*, the output of the BN, the output scale, the mean value, the variance, and the trainable parameters of the scaling operation, respectively.

7 $$ \mu_{m} = \frac{1}{m}\sum\limits_{i = 1}^{m} {x_{i} } $$

8 $$ \sigma_{m}^{2} = \frac{1}{m}\sum\limits_{i = 1}^{m} {(x_{i} - \mu_{m} } )^{2} $$

9 $$ \hat{x}_{i} = \frac{{x_{i} - \mu_{m} }}{{\sqrt {\sigma_{m}^{2} + \varepsilon } }} $$

10 $$ \,y_{i} = \gamma \hat{x}_{i} + \beta . $$

BN and scaling constitute a regularization technology that helps to guarantee that the feature distributions of the training and testing datasets are similar. These can be implemented well after convolution and are a good replacement for the dropout operation. Therefore, this technique makes it possible to train the ultra-deep CS-ResCNN, reduce training time, and improve recognition accuracy.

### Transfer learning

It is well known that the number of samples directly affects the accuracy, depth, and the number of parameters of the CS-ResCNN model. The model is more likely to suffer from an overfitting problem when few samples are involved in training. Because the number of available clinical ophthalmic images is far smaller than the number of available natural images, it is not possible to train the deep CS-ResCNN directly from scratch. Consequently, we can bootstrap the learning process for our ophthalmic images by adopting transfer learning [43, 44] from an existing ResCNN model trained on the large-scale ImageNet datasets [45]. This popular approach can not only make full use of the generic image descriptors from the large sample dataset of natural images but also identify the unique characteristics of ophthalmic images. Moreover, two methods for extending samples, image transformation and horizontal reflection [46], are applied to augment the retro-illumination lens images. Using this scheme, we can train the ultra-deep CS-ResCNN and accelerate convergence on our ophthalmic images.

### Cost-sensitive method and optimization process

The cost factors are included in the loss function of softmax to develop the CS-ResCNN method. Because PCO patients who require surgery are the minority (but more important) class in this study, we discriminatively consider the cost of misclassifying different classes and assign a large cost factor to misclassification of the minority class. Therefore, this technology can simultaneously address the imbalanced dataset problem and minimize the false-negative rates.

Specifically, we randomly selected *m* imbalanced samples to form a set of data sets $\{ (x^{(1)} ,y^{(1)} ), \ldots ,(x^{(m)} ,y^{(m)} )\}$ in one training session, where $x^{\left( i \right)} \in R^{n}$ and $y^{\left( i \right)} \in \left\{ {1, \ldots ,k} \right\}$ indicate the input features and the corresponding labels, respectively. The cross-entropy cost function of our proposed CS-ResCNN method is formalized in Eq. 11:

11 $$ \begin{aligned} J(w) &=  - \frac{1}{m}\left[ {\sum\limits_{i = 1}^{m} {\sum\limits_{j = 1}^{k} {I\left\{ {y^{(i)} = j} \right\} * {\text{C}}\left\{ {y^{(i)} = \hbox{min} \_class} \right\}*\log \frac{{e^{{w_{j}^{T} x^{(i)} }} }}{{\sum\nolimits_{s = 1}^{k} {e^{{w_{s}^{T} x^{(i)} }} } }}} } } \right] \\ &\quad + \frac{\lambda }{2}\sum\limits_{i = 1}^{k} {\sum\limits_{j = 1}^{n} {w_{ij}^{2} } } \\ \end{aligned} $$

where *m*, *w*, *n and k* stand for the size of mini-batch, the trainable parameters, the number of inputs neurons of the softmax classification function and the number of classes, respectively. The $I\left\{ \cdot \right\}$ represents an indicator function (*I*{*a true statement*} = 1 and *I*{*a false statement*} = 0), and $C\{ y^{\left( i \right)} = \hbox{min} \_class\}$ is the cost factor function ($C\{ y^{\left( i \right)} {\text{ is the minority class label}}\} = C_{\hbox{min} }$ and $C\{ y^{\left( i \right)} {\text{ is not the minority class label }}\} = 1$), where *C*_min_ is cost of misclassifying minority and severe PCO patients. By seeking the optimal *C*_min_, we can train the final learning model to have a higher discriminative capability for patients with severe PCO. Furthermore, a weight decay term $\frac{\lambda }{2}\sum\nolimits_{i = 1}^{k} {\sum\nolimits_{j = 1}^{n} {w_{ij}^{2} } }$ is applied to penalize larger values of the trainable weights. Eventually, the mini-batch gradient descent method (mini-batch-GD) [47] is adopted to minimize the cross-entropy function *J*(*w*). To obtain the optimal parameters of the CS-ResCNN in this process, we compute the derivative of the cross-entropy function *J*(*w*) as shown in Eq. 12:

12 $$\begin{aligned} \nabla_{{w_{j} }} J(w) &= - \frac{1}{m}\sum\limits_{i = 1}^{m} {\left[ {C\left\{ {y^{(i)} = {\text{min-class}}} \right\}*x^{(i)} *(I\{ y^{(i)} = j\} - p(y^{(i)} = j|x^{(i)} ;w))} \right]} \\ &\quad+ \lambda w_{j} \end{aligned} $$

Moreover, the parameters of all the previous layers can be achieved using the chain rule of the back-propagation (BP) method. By optimizing the cross-entropy function *J*(*w*), we can achieve the most appropriate parameter weight $w^{*}$ as shown in Eq. 13.

13 $$ w^{*} = \arg \mathop {\hbox{min} }\limits_{w} J(w) $$

### Experimental environment

In this study, the CS-ResCNN was implemented using the Caffe toolbox [48] and trained in parallel on four NVIDIA TITAX X GPUs. The size of mini-batch was set to 64 on each GPU to accelerate parameter convergence. The initial learning rate and the maximum number of iterations were set to 0.001 and 2000, respectively. Then, the learning rate was successively reduced to one-tenth of the original value in steps of 500 iterations. The settings of these super parameters are appropriate for our imbalanced datasets to guarantee better performance and prevent divergence.

## Results and discussion

### Automatic localization for preprocessing

**Fig. 3:**
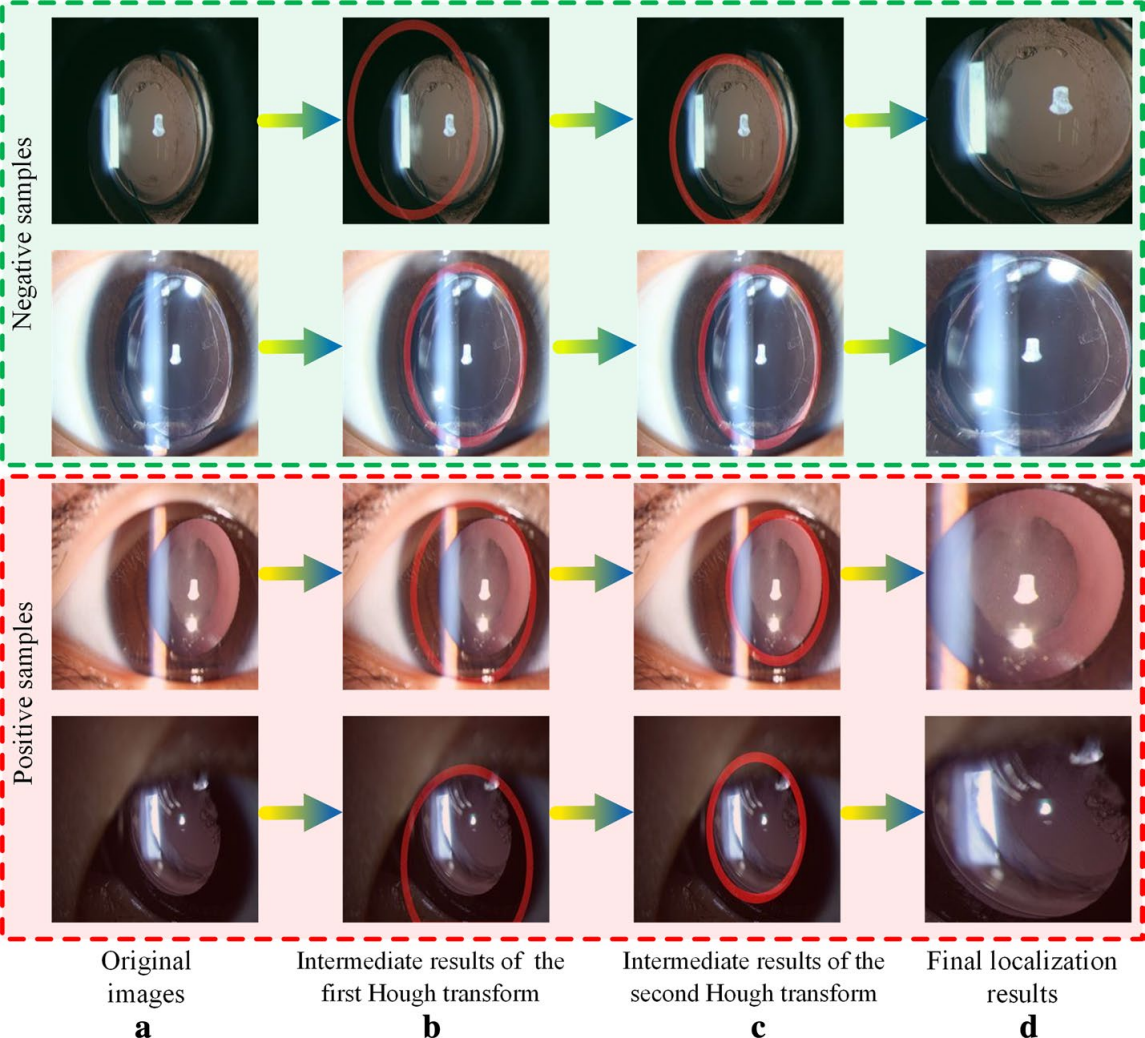
Examples of the automatic lens localization process. **a** Four representative positive and negative samples from the original images. **b**, **c** The intermediate localization results of twice-applied Canny detections and Hough transformations. **d** Final localization results

Twice-applied Canny detections and Hough transformations [36, 37] were employed for image preprocessing to eliminate noise. Four typical positive and negative cases are presented to illustrate the effectiveness of automatic lens localization (Fig. 3). By twice transforming the original retro-illumination images (Fig. 3a), we achieved the intermediate results shown in Fig. 3b, c, where the bold red circles intuitively demonstrate the effect of localization. The localization result in Fig. 3c indicates that we can identify the lens area precisely and filter out most of the surrounding noise. Finally, we cut the images along the red borderlines to form the dataset used with the CS-ResCNN model (Fig. 3d).

Furthermore, the prevalent intersection-over-union (IoU) [49] indicator of the image segmentation algorithms was employed to evaluate the accuracy of the Canny detection and Hough transformation method. The IoU indicator is formalized as Eq. 14, where *B*_*p*_ and *B*_*gt*_ represent the prediction and ground truth bounding box of crystalline lens, respectively. $B_{p} \cap B_{gt}$ denotes the intersection of the prediction and ground truth bounding boxes and $B_{p} \cup B_{gt}$ is their union. Specifically, 100 samples were randomly selected from the whole dataset of ocular images and the boundaries of the crystalline lens were manually labelled by a senior ophthalmologist. We calculated the mean value of IoUs over these 100 samples and achieved a satisfactory segmentation result (82.93%).

14 $$ IoU = \frac{{area(B_{p} \cap B_{gt} )}}{{area(B_{p} \cup B_{gt} )}} $$

### Effectiveness analysis of deep features

**Fig. 4:**
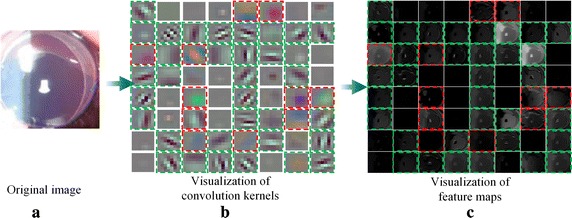
Visualization of first-layer convolution kernels and feature maps for the CS-ResCNN method. The green and red squares denote the captured edges and color characteristics, respectively. **a** Original retro-illumination image. **b** The 64 convolution kernels with dimensions of 7 × 7 projected into pixel space. **c** The 64 feature maps with dimensions of 56 × 56

Hierarchical visualization technology [48, 50, 51] and t-distributed stochastic neighbor embedding (t-SNE) [52] were employed to intuitively analyze the characteristics of the extracted hierarchical features. It is well known that convolutional kernels can effectively capture and generate biological features such as edges or colors [50, 51]. Using the hierarchical visualization method, we visualized the first-layer convolution kernels (Fig. 4b), in which the 11 green and 33 red squares served as edge and color extractors, respectively. Correspondingly, we obtained representative feature maps (Fig. 4c) from the original image (Fig. 4a). The visualization results support the idea that most of the extracted low-level feature maps are meaningful and can effectively represent the original image.

**Fig. 5:**
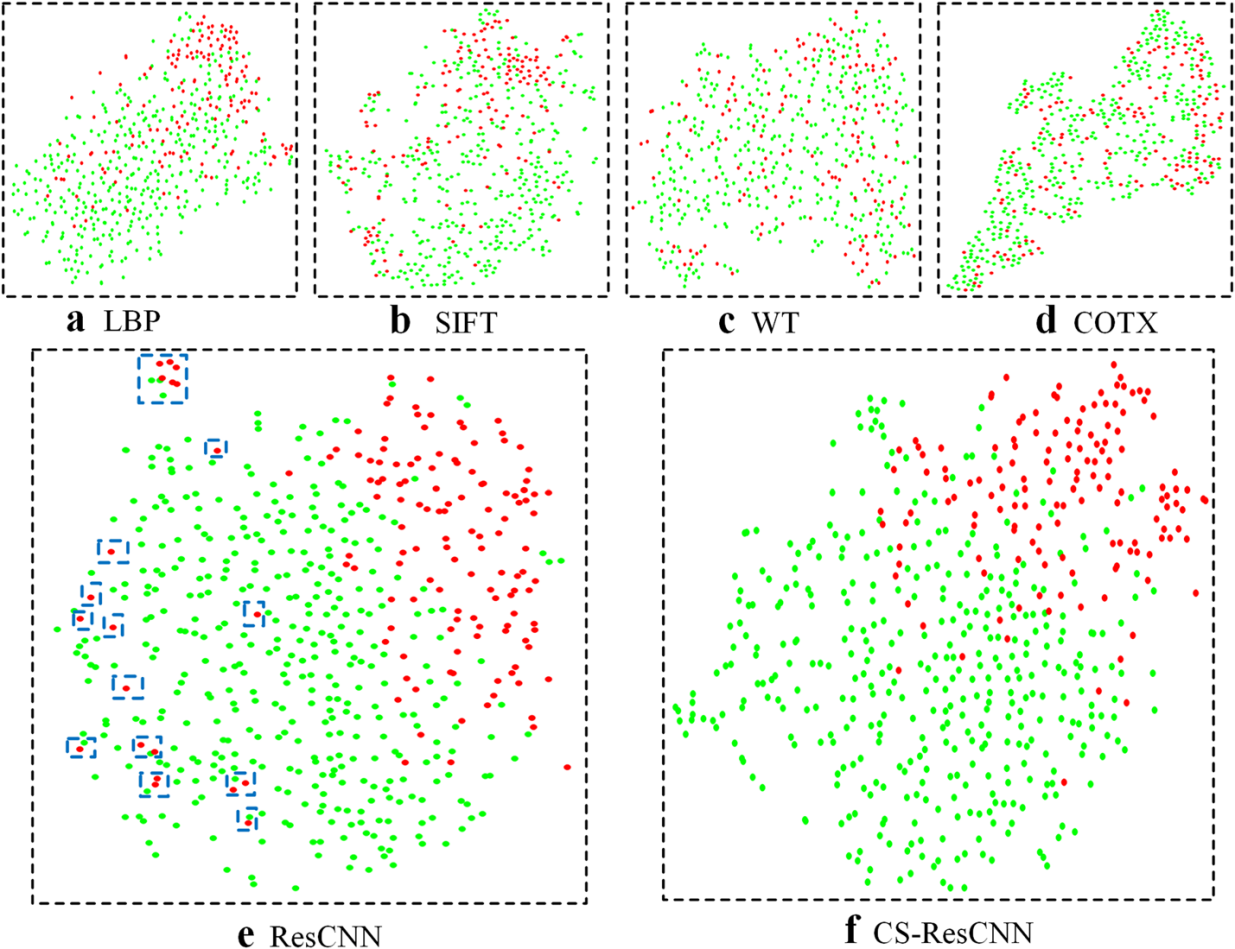
CS-ResCNN feature maps and representative conventional features using t-SNE. **a**–**f** Two-dimensional maps of LBP, SIFT, WT, COTE, ResCNN and CS-ResCNN methods, respectively. The red and green dots represent positive and negative samples. t-SNE, t-distributed stochastic neighbor embedding; CS-ResCNN, cost-sensitive residual convolutional neural network; WT, wavelet transformation; SIFT, scale-invariant feature transform; LBP, local binary pattern; COTE, color and texture features

We further applied the t-SNE method to investigate whether the extracted high-level features were discriminative. High-level features were mapped into two-dimensional space to determine their discrimination ability. Visualized maps of the high-level features extracted using four conventional methods (LBP: local binary pattern, WT: wavelet transformation, SIFT: scale-invariant feature transform, and COTE: color and texture features) and two deep learning methods (CS-ResCNN and native ResCNN) are displayed separately in Fig. 5. The red and green points denote the positive and negative samples, respectively. The discrimination ability of the conventional features is quite weak and obviously inferior to that of the two deep learning features. Moreover, using the cost-sensitive method, the separability of the CS-ResCNN features was markedly improved compared with ResCNN. Specifically, the proportion of very scattered positive samples (the blue rectangles in Fig. 5) that are easily misdiagnosed was significantly reduced. This result suggests that the high-level features of the CS-ResCNN method can be used to more easily identify the positive samples.

In addition, we explored the effectiveness of another cost-sensitive method, threshold-moving method [22, 53], which adjusts the output threshold toward low cost classes to ensure that high cost classes are harder to be misclassified. This method may also be effective for imbalanced dataset when class weights were set properly. However, the high-level features of the threshold-moving method were inappropriate for imbalanced dataset because the class weights were only involved in the testing process rather than the training process (e.g., the ResCNN method).

### Exploring the range of the cost factor

**Fig. 6:**
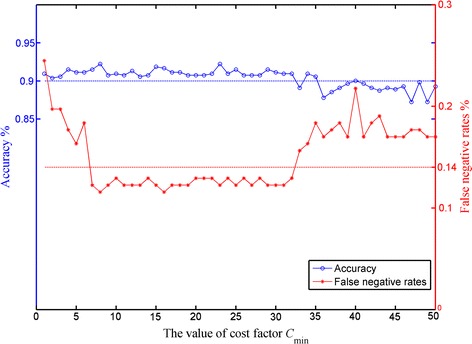
The accuracy and FNR curves with the value of the cost factor *C*_min_. The blue and red curves represent the trends of FNR and accuracy with the cost factor *C*_min_, respectively where the two dashed lines at 0.9 and 0.14 are provided for reference. FNR, false negative rate

We used a grid-search procedure to determine the adaptive range of the cost factor (details in “Methods”). We set the cost of misclassified negative and positive samples to one and *C*_min_, respectively; a correct classification is set to zero (Table 1). Specifically, we set the cost factor *C*_min_ within the range [1–50] with a step size of one. Accuracy and FNR (1-sensitivity) curves were plotted for evaluating the trained classifiers (Fig. 6). Two dashed lines are shown at 0.14 and 0.9 of the vertical axis for comparison purposes. Our model became unstable when *C*_min_ is below 7 or above 32, which suggests that exceeding those limits might not be appropriate in this situation. The FNR fluctuation is subtle and the classifier reaches an accuracy greater than 90% when the cost factor is within a relatively wide interval [7–32]. This satisfactory stability is mainly contributed by the applications of transfer learning, cost-sensitive, batch normalization and residual connection techniques. It also indicates that the CS-ResCNN method is not sensitive to the cost factor. Given this identified scope, we set the cost factor to twelve in subsequent experiments.

**Table 1:** The cost factors and data distribution in imbalanced retro-illumination images

Actual class	Predicted class	
	Majority/negative	Minority/positive
Majority/negative (1970)	0	1
Minority/positive (735)	*C*_min_	0

The numbers of positive and negative samples in the dataset were 735 and 1970, respectively. The cost factors for misclassifying positive and negative samples were *C*_min_ and one respectively while the cost factor for correct classification was zero.

Similarly, the grid-search procedure was employed to analyze the effective weights’ interval in the threshold-moving method. However, the appropriate weight of misclassifying positive is within a relatively narrow interval [4–6]. Specifically, when the weights of the misclassifying positive and negative samples were only set to four and one, respectively, the performance of the threshold-moving method (ACC: 91.18%, SPC: 92.50%, SEN: 87.62%, F1_M: 84.06%, and G_M: 89.99%) was almost equal to that of CS-ResCNN method. Otherwise, the performance of threshold-moving method will be degraded severely.

### Parameters setting and classifiers selection for conventional methods

To evaluate the performance and feasibility of the CS-ResCNN model in detail, we employed four representative feature extraction methods [27, 29] (LBP, WT, SIFT, and COTE), two excellent classifiers [support vector machine (SVM) and random forest (RF)] and three data-level methods [18, 19, 22] [the synthetic minority oversampling technique (SMOTE), borderline-SMOTE (BSMOTE) and under-sampling (UNDER)] for comparison. To achieve the optimal performance of the conventional methods, we firstly presented detailed parameters for classifiers, feature extraction methods and data-level methods as shown in Table 2. Specifically, we chose the parameters of the feature extraction methods and classifiers based on our previous research [27–29]. For the data-level methods (SMOTE, borderline-SMOTE and UNDER), we mainly referred to the previous studies [18, 19, 22] and their open source codes. Moreover, we conducted extensive parameter adjustment experiments and performance comparison to ensure the usage of optimal parameters for the ophthalmic images. Based on the experimental results, the performance of the RF classifier is superior to that of the SVM classifier, which is consistent with the previous study [54]. Therefore, the RF was selected for the final comparative experiments and the results of the SVM were also presented in Additional file 1: Table S1.

**Table 2:** The relevant parameters of conventional methods

Classifiers	Features extraction methods [27, 29]	Data-level methods [18, 19, 22]
Random forest (RF): number of trees = 300 Support vector machine (SVM): linear kernel function	Color and texture features (COTX): gray tone spatial dependence matrices: d = 1; gray gradient co-occurrence matrices: Lg = 10	SMOTE and BorSMOTE: nearest neighbors k = 5, the ratio of positive and negative samples r = 1 Under-sampling: the ratio of positive and negative samples r = 1
	Local binary pattern (LBP): P = 9	
	Wavelet transformation (WAVE): two level wavelet transformation, Haar wavelet	
	Scale-invariant feature transform (SIFT)	

### Performance comparisons with conventional methods

**Fig. 7:**
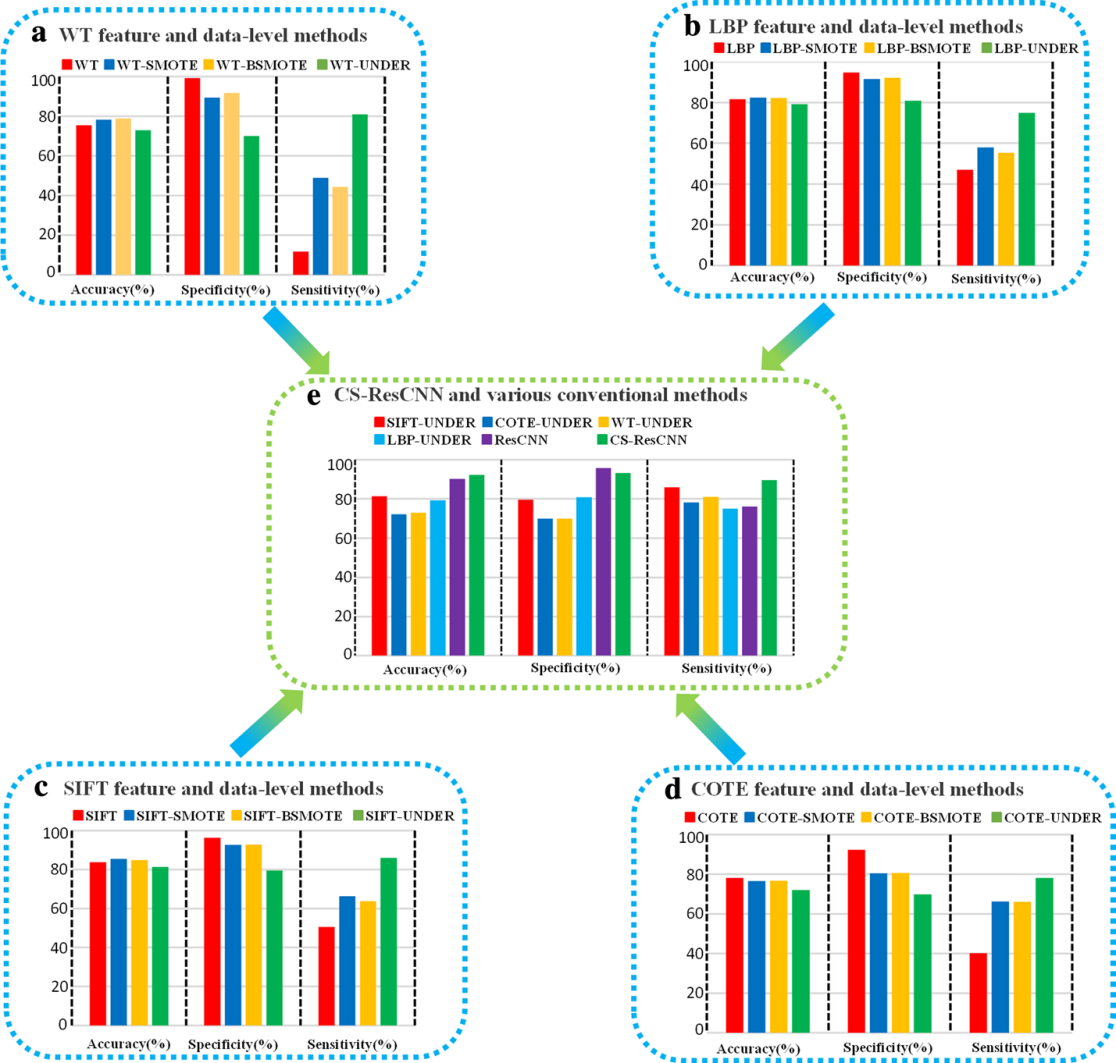
Performance comparison of the CS-ResCNN method and various conventional methods. Two sets of deep learning methods and 16 sets of conventional methods were evaluated using accuracy, sensitivity and specificity indicators. **a**–**d** The four conventional methods WT, LBP, SIFT and COTE, respectively, compared with three data-level methods; **e** the CS-ResCNN method and five representative conventional methods (ResCNN, SIFT-UNDER, COTE-UNDER, WT-UNDER and LBP-UNDER). CS-ResCNN, cost-sensitive residual convolutional neural network; ResCNN, native residual convolutional neural network; WT, wavelet transformation; LBP, local binary pattern; SIFT, scale-invariant feature transform; COTE, color and texture features; SMOTE, synthetic minority over-sampling technique; BSMOTE, borderline-SMOTE; UNDER, under-sampling

After applying *K*-fold cross-validation (*K* = 5), we obtained a total of 18 comparative experiment results. We calculated the accuracy (ACC), sensitivity (SEN) and specificity (SPC) indicators for the results, which included 16 sets from conventional methods (Fig. 7a–d) and two from deep learning methods (Fig. 7e). The means and standard deviations of other detailed quantitative performance indicators were also calculated (Table 3).

**Table 3:** Quantitative evaluation of the CS-ResCNN method and various conventional methods

Methods	ACC (%)	SPC (%)	SEN (%)	F1_M (%)	G_M (%)	AUC (%)
WT	75.49 (0.70)^a^	99.29 (0.90)	11.70 (1.55)	20.57 (2.42)	34.02 (2.26)	82.50 (1.93)
WT-SMOTE	78.34 (1.35)	89.34 (0.97)	48.84 (2.52)	55.06 (2.81)	66.05 (2.04)	83.18 (2.05)
WT-BSMOTE	78.85 (1.48)	91.73 (0.79)	44.35 (3.48)	53.24 (3.72)	63.75 (2.79)	83.89 (2.37)
WT-UNDER	72.98 (2.07)	70.00 (2.63)	80.95 (3.37)	61.96 (2.36)	75.25 (2.02)	83.40 (2.38)
LBP	81.70 (0.68)	94.67 (0.78)	46.94 (3.15)	58.18 (2.35)	66.63 (2.06)	86.04 (1.66)
LBP-SMOTE	82.40 (1.37)	91.52 (1.37)	57.96 (2.38)	64.16 (2.58)	72.82 (1.76)	86.78 (1.81)
LBP-BSMOTE	82.18 (1.07)	92.18 (1.13)	55.37 (1.77)	62.81 (2.03)	71.44 (1.36)	86.57 (1.80)
LBP-UNDER	79.22 (2.16)	80.81 (1.82)	74.97 (4.45)	66.22 (3.50)	77.81 (2.80)	86.33 (1.92)
SIFT	83.81 (0.91)	96.24 (1.05)	50.48 (2.12)	62.88 (2.01)	69.69 (1.47)	91.14 (1.44)
SIFT-SMOTE	85.43 (2.20)	92.59 (1.24)	66.26 (4.94)	71.16 (4.56)	78.30 (3.37)	91.34 (1.32)
SIFT-BSMOTE	84.88 (1.21)	92.74 (1.44)	63.81 (2.86)	69.63 (2.32)	76.91 (1.74)	91.31 (1.34)
SIFT-UNDER	81.29 (1.57)	79.54 (1.85)	85.99 (2.07)	71.43 (2.02)	82.69 (1.52)	91.29 (1.11)
COTE	78.15 (1.10)	92.34 (1.26)	40.14 (3.97)	49.89 (3.47)	60.81 (2.91)	82.19 (2.34)
COTE-SMOTE	76.60 (1.07)	80.46 (0.74)	66.26 (3.35)	60.59 (2.26)	72.99 (1.92)	81.41 (1.68)
COTE-BSMOTE	76.75 (1.32)	80.71 (0.65)	66.12 (3.83)	60.68 (2.72)	73.03 (2.28)	80.98 (1.61)
COTE-UNDER	72.09 (1.52)	69.85 (1.65)	78.10 (3.74)	60.32 (2.24)	73.83 (1.93)	80.87 (1.73)
ResCNN	90.22 (0.88)	95.80 (1.23)	76.05 (3.21)	81.41 (1.74)	85.34 (1.59)	96.26 (0.73)
*CS*-*ResCNN*	*92.24 (1.30)*	*93.19 (1.73)*	*89.66 (2.86)*	*86.00 (2.27)*	*91.39 (1.49)*	*97.11 (0.59)*

The random forest classifier is employed for the conventional methods. ResCNN, residual convolutional neural network; CS-ResCNN, cost-sensitive residual convolutional neural network; WT, wavelet transformation; LBP, local binary pattern; SIFT, scale-invariant feature transform; COTE, color and texture features; SMOTE, synthetic minority over-sampling technique; BSMOTE, borderline-SMOTE; UNDER, under-sampling; ACC, accuracy; SPC, specificity; SEN, sensitivity; F1_M, F1-measure; G_M, G-mean; AUC, area under the receiver operating characteristic curve

Italics represents the best value in all methods

^a^Mean (standard deviation)

First, the conventional feature methods without data-level technology have the same fatal flaws: low accuracy and sensitivity (the red bar in Fig. 7a–d). In particular, the sensitivity of the WT method is less than 12%—and the best SIFT performance is no more than 50.48%. These experimental results confirm that the conventional methods do not consider the class imbalance problem; consequently, their recognition rates are biased toward the majority class and tend to overlook the minority class.

After applying data-level processing, the SEN results for almost all conventional features combined with SMOTE, borderline-SMOTE or under-sampling methods were significantly enhanced compared with the original features (Fig. 7a–d). However, this improvement comes at the expense of a reduction in SPC. For example, as the SEN of WT-SMOTE increases from 11.70 to 48.84%, its SPC diminishes from 99.29 to 89.34% (the blue and red bars in Fig. 7a); correspondingly, the trends of the SIFT-UNDER and COTE-SMOTE methods are similar (the green and blue bars in Fig. 7c, d). From the overall comparisons, the under-sampling method is superior to the over-sampling methods (SMOTE and borderline-SMOTE); the performance of the SMOTE and the borderline-SMOTE is almost equivalent. Furthermore, these data-level methods provide inferior results in terms of other quantitative measures such as the F1-measure, G-mean and AUC (Table 3), and they cannot be implemented effectively in clinical applications.

Finally, we presented the results of the CS-ResCNN method (Fig. 7e) compared with four of the relatively superior data-level methods (SIFT-UNDER, COTE-UNDER, WT-UNDER and LBP-UNDER) selected from Fig. 7a–d. The CS-ResCNN method is far superior to the conventional features and the data-level methods with respect to all evaluation indicators (the green bar in Fig. 7e). Furthermore, compared with native ResCNN, the CS-ResCNN method significantly enhances the overall performance of the model, especially regarding the SEN, F1-measure and G-mean, which improved by more than 13.6, 4.5 and 6%, respectively (Fig. 7e and Table 3). Meanwhile, the CS-ResCNN maintains the SPC within an acceptable range (a 2.6% reduction). Overall, our proposed method yields superior results in terms of ACC (92.24%), SPC (93.19%), SEN (89.66%), the F1-measure (86.00%), the G-mean (91.39%), and the AUC (97.11%) (Table 3). The superior performance of the CS-ResCNN method indicates that it can provide an effective solution for the imbalanced ophthalmic dataset problem and successfully classify PCO after pediatric cataract surgery.

**Fig. 8:**
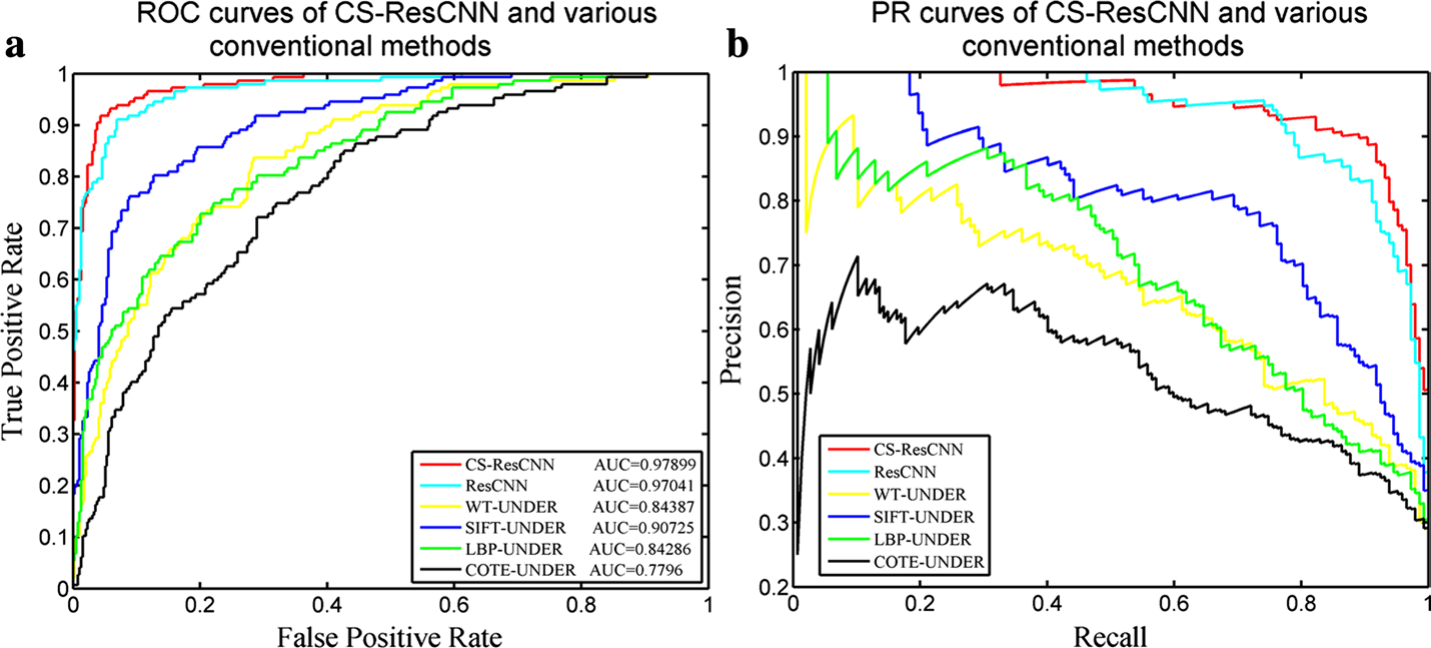
The ROC and PR curves for the CS-ResCNN method and representative conventional methods. **a** The ROC curves and AUC values for the CS-ResCNN method and five compared methods: ResCNN, SIFT-UNDER, COTE-UNDER, WT-UNDER and LBP-UNDER. **b** The PR curves for the CS-ResCNN method and the five compared methods. ROC, receiver operating characteristic curve; AUC, area under the ROC curve; PR, precision–recall; CS-ResCNN, cost-sensitive residual convolutional neural network; ResCNN, native residual convolutional neural network; UNDER, under-sampling; WT, wavelet transformation; LBP, local binary pattern; SIFT, scale-invariant feature transform; COTE, color and texture features

Furthermore, we plotted the ROC and PR curves to investigate the performance of the CS-ResCNN method in more detail compared with other methods (Fig. 8a, b). The upper-left corner of the ROC curve and the upper-right corner of the PR curve indicate a superior classifier. From high to low performance, the classifiers are CS-ResCNN, ResCNN, SIFT-UNDER, LBP-UNDER, WT-BSMOTE and COTE-UNDER, respectively. These results indicate that the CS-ResCNN method considerably outperforms the other conventional methods and native ResCNN. Although the native ResCNN curves are close to the CS-ResCNN curves, the CS-ResCNN curves are smoother, and our proposed method performs better. These results also indicate the superiority of deep learning methods in current image processing tasks.

### Exploring the effectiveness of the combinations of cost-sensitive and data-level methods

Since the data-level methods and cost-sensitive are two powerful techniques for addressing the imbalanced dataset from different perspectives. It is expected that the combinations of these two approaches could further enhance the recognition ability of the model. Generally, the data-level methods are used to process the features of the images rather than the images. Therefore, we extracted the high-level features from the ResCNN and CS-ResCNN, and then employed the over-sampling and under-sampling technologies to balance the proportions of the positive and negative samples. Also, the RF classifier was employed for these balanced features. Finally, a total of eight methods were performed and compared in detail (Table 4). From the comparative experiments, we obtained three meaningful conclusions. First, the under-sampling method is superior to the over-sampling methods (SMOTE and borderline-SMOTE). And the performance of the SMOTE and the borderline-SMOTE is almost equivalent. These results are consistent with the conclusion in the conventional methods. Second, the combinations of the cost-sensitive and data-level methods are better than those using only data-level methods. Third, the combination of the cost-sensitive and under-sampling method is almost equal to the CS-ResCNN method. However, the efficiency of the CS-ResCNN is optimal because no extra operation is required. The above comparative experiments and analyses indicate that these combinations of cost-sensitive and data-level methods do not further improve the performance of the model.

**Table 4:** Quantitative evaluation of the combinations of cost-sensitive and data-level methods using CNN features

Methods	ACC (%)	SPC (%)	SEN (%)	F1_M (%)	G_M (%)	AUC (%)
ResCNN	90.22 (0.88)^a^	95.80 (1.23)	76.05 (3.21)	81.41 (1.74)	85.34 (1.59)	96.26 (0.73)
ResCNN + SMOTE	90.98 (1.07)	94.72 (1.34)	80.95 (3.50)	82.97 (2.05)	87.54 (1.75)	96.24 (0.84)
ResCNN + BSMOTE	90.76 (1.40)	95.48 (1.54)	78.10 (2.94)	82.12 (2.55)	86.34 (1.79)	96.27 (0.87)
ResCNN + UNDER	90.02 (1.68)	90.91 (1.71)	87.62 (3.71)	82.67 (2.82)	89.23 (2.14)	96.27 (0.80)
*CS*-*ResCNN*	*92.24 (1.30)*	*93.19 (1.73)*	*89.66 (2.86)*	*86.00 (2.27)*	*91.39 (1.49)*	*97.11 (0.59)*
CS-ResCNN + SMOTE	92.35 (1.01)	95.08 (0.93)	85.03 (4.91)	85.74 (2.21)	89.88 (2.31)	97.36 (0.70)
CS-ResCNN + BSMOTE	92.01 (0.86)	95.48 (1.04)	82.72 (3.78)	84.89 (1.83)	88.85 (1.80)	97.22 (0.65)
CS-ResCNN + UNDER	91.83 (0.85)	92.79 (1.74)	89.25 (3.80)	85.58 (1.42)	90.97 (1.41)	97.35 (0.61)

ResCNN, residual convolutional neural network; CS-ResCNN, cost-sensitive residual convolutional neural network; SMOTE, synthetic minority over-sampling technique; BSMOTE, borderline-SMOTE; UNDER, under-sampling; CS-ResCNN + SMOTE, the combination of CS-ResCNN and SMOTE methods; CS-ResCNN + BSMOTE, the combination of CS-ResCNN and BSMOTE methods; CS-ResCNN + BSMOTE, the combination of CS-ResCNN and UNDER methods

Italic represent the best value in all methods

^a^Mean (standard deviation)

### Convergence analysis of the CS-ResCNN model

**Fig. 9:**
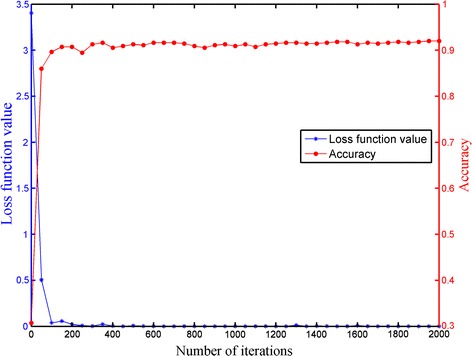
The accuracy and loss function value curves with the iterations. The blue and red curves represent the trends of the loss function value and accuracy on testing dataset, respectively

We also analyzed the convergence of the CS-ResCNN model in detail under limited training time. We performed a total of 2000 training sessions and calculated one accuracy and loss function value on the testing dataset every 50 iterations. As shown in Fig. 9, the loss function value and accuracy rate of the testing dataset changed dramatically at first and then stabilized after 500 iterations, showing that our model reaches good convergence on the imbalanced dataset problem. This satisfactory performance is mainly contributed by the applications of these techniques, including the transfer learning, data augmentation, the batch normalization and non-saturating ReLU function, which can effectively avoid over-fitting problem and ensure the generalization capability of the model.

### Web server for clinical applications

We developed a web-based CAD system for patients and ophthalmologists at Zhongshan Ophthalmic Center at Sun Yat-sen University to promote future clinical application use of our model. The website provides detailed specifications and imposes no access restrictions. Users only need to click one button to upload the test retro-illumination images; then, our software can simultaneously localize the lens region of interest (ROI) and perform ophthalmic disease diagnosis. As implemented, the software can predict multiple images at a time. We hope that our work will help to provide high-quality medical care with personalized treatment recommendations for patients in less-developed areas where advanced medical devices and well-trained doctors are scarce. After a period of clinical application, we are able to upgrade the model to further enhance its accuracy and reliability with large amounts of accumulated datasets. This process takes only half an hour using four GPUs.

### Code availability

The source code of the CS-ResCNN for retro-illumination images is available from Github: https://github.com/Ophthalmology-CAD/retro-illumination-images.

### CAD software availability

The website of the computer-aided diagnosis software is available at http://www.cc-cruiser.com:5008/retro_illumination_images_prediction.

## Conclusions and future work

In this paper, we proposed a feasible and automatic approach based on our CS-ResCNN model to effectively address the problem of misclassifications resulting from imbalanced ophthalmic images datasets. Our method demonstrates high performance and robustness within an adaptive cost factor range. Qualitative analyses of the visualized results illustrate that the features extracted from the CS-ResCNN are meaningful and discriminative, and quantitative assessments indicate that the CS-ResCNN model not only maintains an acceptable SPC range but also significantly boosts the ACC, SEN, F1-measure and G-mean indicators. The results of abundant experimental comparisons revealed that our proposed CS-ResCNN method outperforms both other conventional features and data-level methods (SMOTE, borderline-SMOTE and under-sampling) as well as the native CNN approach.

In the future, we will explore and compare additional potential algorithms such as U-Net or Faster R-CNN for the segmentation and grading of the ophthalmic images. Then, we will investigate how to integrate multi-source images and multiple deep learning models to further enhance the performance of the CS-ResCNN method. Moreover, our cost-sensitive pattern can be applied and serve as an important reference for other imbalanced medical classification studies while smoothing the path for adopting artificial intelligence techniques in clinical applications.

## Additional file

**Additional file 1: Table S1.** Quantitative evaluation of the CS-ResCNN method and various conventional methods with SVM classifier.

## Abbreviations

CNN: convolutional neural network
CS-ResCNN: cost-sensitive residual convolutional neural network
ResCNN: native residual convolutional neural network
CAD: computer-aided diagnosis
BN: batch normalization
ReLU: rectified linear unit
mini-batch-GD: mini-batch gradient descent method
BP: back-propagation
t-SNE: t-distributed stochastic neighbor embedding
WT: wavelet transformation
LBP: local binary pattern
SIFT: scale-invariant feature transform
COTE: color and texture features
SMOTE: synthetic minority over-sampling technique
BSMOTE: borderline-SMOTE
UNDER: under-sampling
ACC: accuracy
SPC: specificity
SEN: sensitivity
F1_M: F1-measure
G_M: G-mean
ROC: receiver operating characteristic curve
AUC: area under the ROC curve
PR: precision–recall
ROI: region of interest
FNR: false negative rate
FPR: false positive rate
Nd-YAG: neodymium-doped yttrium aluminum garnet
PCO: posterior capsular opacification
CCPMOH: Childhood Cataract Program of the Chinese Ministry of Health
