# Supplementary material for: Automatic diagnosis of imbalanced ophthalmic images using a cost-sensitive deep convolutional neural network
Source: Biomed Eng Online. 2017 Nov 21;16:132. doi: 10.1186/s12938-017-0420-1 (PMC5697161; doi:10.1186/s12938-017-0420-1)
Supplement: Supplementary file 1 — Additional file 1: Table S1. Quantitative evaluation of the CS-ResCNN method and various conventional methods with SVM classifier. [file 12938_2017_420_MOESM1_ESM.docx]

# Supplementary Tables

**Table S1. Quantitative evaluation of the CS-ResCNN method and various conventional methods.**

| Method | ACC (%) | SPC (%) | SEN (%) | F_M (%) | G_M (%) | AUC (%) |
| --- | --- | --- | --- | --- | --- | --- |
| WT | 72.75(0.17)^§^ | 99.80(0.45) | 0.27(0.61) | 0.52(1.17) | 2.32(5.19) | 49.40(7.59) |
| WT-SMOTE | 49.13(7.96) | 46.75(15.8) | 55.51(15.9) | 36.79(5.09) | 48.81(6.55) | 56.01(6.15) |
| WT-BSMOTE | 46.69(10.3) | 39.49(20.7) | 65.99(18.2) | 39.92(2.84) | 45.95(15.8) | 61.59(15.5) |
| WT-UNDER | 69.65(4.68) | 92.94(10.0) | 7.21(10.1) | 9.07(10.61) | 17.95(18.3) | 54.17(3.19) |
| LBP | 74.38(1.14) | 92.49(2.77) | 25.85(5.16) | 35.16(4.60) | 48.64(4.45) | 74.40(3.37) |
| LBP-SMOTE | 72.01(1.75) | 78.93(2.98) | 53.47(2.29) | 50.96(1.26) | 64.93(0.89) | 74.40(3.37) |
| LBP-BSMOTE | 71.39(2.31) | 76.35(3.42) | 58.10(1.84) | 52.51(1.94) | 66.57(1.38) | 74.40(3.37) |
| LBP-UNDER | 66.99(2.41) | 67.01(3.48) | 66.94(2.57) | 52.46(2.01) | 66.94(1.81) | 73.32(2.00) |
| SIFT | 83.33(2.10) | 88.83(2.14) | 68.57(5.62) | 69.04(4.04) | 77.99(3.26) | 89.08(1.76) |
| SIFT-SMOTE | 64.62(2.04) | 60.46(2.18) | 75.78(4.21) | 53.78(2.53) | 67.66(2.32) | 75.52(2.98) |
| SIFT-BSMOTE | 62.22(0.90) | 55.43(2.10) | 80.41(4.57) | 53.60(1.53) | 66.71(1.14) | 76.91(2.98) |
| SIFT-UNDER | 74.75(1.64) | 74.97(4.12) | 74.15(5.75) | 61.46(1.20) | 74.43(1.20) | 82.03(1.50) |
| COTE | 72.87(0.42) | 99.80(0.21) | 0.68(1.52) | 1.31(2.92) | 3.68(8.24) | 61.86(5.58) |
| COTE-SMOTE | 50.17(13.0) | 43.10(29.7) | 69.12(32.3) | 40.89(8.76) | 46.73(6.45) | 62.86(5.19) |
| COTE-BSMOTE | 53.16(13.5) | 52.28(31.3) | 55.51(35.2) | 35.86(11.3) | 43.95(11.5) | 58.23(4.67) |
| COTE-UNDER | 53.60(12.1) | 51.07(28.6) | 60.41(33.0) | 38.90(8.67) | 48.29(3.23) | 63.75(14.1) |
| ResCNN | 90.22(0.88) | 95.80(1.23) | 76.05(3.21) | 81.41(1.74) | 85.34(1.59) | 96.26(0.73) |
| CS-ResCNN | *92.24(1.30)* | *93.19(1.73)* | *89.66(2.86)* | *86.00(2.27)* | *91.39(1.49)* | *97.11(0.59)* |

The support vector machine classifier is employed for the conventional methods. Notes: ResCNN: residual convolutional neural network; CS-ResCNN: cost-sensitive residual convolutional neural network; WT: wavelet transformation; LBP: local binary pattern; SIFT: scale-invariant feature transform; COTE: color and texture features; SMOTE: synthetic minority over-sampling technique; BSMOTE: borderline-SMOTE; UNDER: under-sampling; ACC: accuracy; SPC: specificity; SEN: sensitivity; F_M: F1-measure; G_M: G-mean; AUC: area under the receiver operating characteristic curve; ^§^Mean (Standard Deviation).
